# Noise reduction in single time frame optical DNA maps

**DOI:** 10.1371/journal.pone.0179041

**Published:** 2017-06-22

**Authors:** Paola C. Torche, Vilhelm Müller, Fredrik Westerlund, Tobias Ambjörnsson

**Affiliations:** 1 Department of Astronomy and Theoretical Physics, Lund University, Lund, Sweden; 2 Department of Biology and Biological Engineering, Chalmers, University of Technology, Gothenburg, Sweden; Imperial College London, UNITED KINGDOM

## Abstract

In optical DNA mapping technologies sequence-specific intensity variations (DNA barcodes) along stretched and stained DNA molecules are produced. These “fingerprints” of the underlying DNA sequence have a resolution of the order one kilobasepairs and the stretching of the DNA molecules are performed by surface adsorption or nano-channel setups. A post-processing challenge for nano-channel based methods, due to local and global random movement of the DNA molecule during imaging, is how to align different time frames in order to produce reproducible time-averaged DNA barcodes. The current solutions to this challenge are computationally rather slow. With high-throughput applications in mind, we here introduce a parameter-free method for filtering a single time frame noisy barcode (snap-shot optical map), measured in a fraction of a second. By using only a single time frame barcode we circumvent the need for post-processing alignment. We demonstrate that our method is successful at providing filtered barcodes which are less noisy and more similar to time averaged barcodes. The method is based on the application of a low-pass filter on a single noisy barcode using the width of the Point Spread Function of the system as a unique, and known, filtering parameter. We find that after applying our method, the Pearson correlation coefficient (a real number in the range from -1 to 1) between the single time-frame barcode and the time average of the aligned kymograph increases significantly, roughly by 0.2 on average. By comparing to a database of more than 3000 theoretical plasmid barcodes we show that the capabilities to identify plasmids is improved by filtering single time-frame barcodes compared to the unfiltered analogues. Since snap-shot experiments and computational time using our method both are less than a second, this study opens up for high throughput optical DNA mapping with improved reproducibility.

## Introduction

In *optical DNA mapping* technologies, single DNA molecules are characterized by using fluorescent, sequence-specific, labeling. The fluorescently stained DNA molecules are stretched using surface adsorption or in nanochannels and visualized using fluorescence microscopy. Optical DNA mapping allows to measure coarse intensity maps of DNA sequences, with resolution on the order of 1 kilobasepairs (kbp) [1]. Optical maps are often used as a complement to DNA sequencing, serving as a scaffold for assembling and validation [2–10]. Optical maps are also used in the characterization of structural variations (inversions, deletions, translocations, duplications, insertions) [11–16], which have shown in many studies correlation to human diseases [17–22]. Other applications are the detection of antibiotic resistance in plasmids from bacteria [1, 23–26], and rapid identification of bacterial species [27–31].

There are several types of fluorescent labeling techniques used in optical DNA mappings. The labeling methods may be roughly classified into two categories: (i) sparse labeling, and (ii) dense labeling. The word “label” is here used in its broadest sense and can represent a fluorescent signal, or a dark region, between fluorescent labels, for instance. Category (i) contains cases where each label can be visually (and algorithmically) identified in the optical map. Examples of sparsely labeled maps include restriction enzyme cut DNA fragmentation maps [32, 33]. In this method, fluorescently labeled DNA molecules are stretched, typically using surface adsorption, and cut at specific sequence dependent positions. When visualized in a microscope, the cut positions along the DNA appear as dark regions (the labels) in between bright regions (intact DNA). Another example of category (i) barcodes is sparse enzymatically labeled optical maps [34, 35] where the fluorescence of sequence-specifically bound molecules serve as labels. For type (ii) labeling, the sequence-dependent DNA fingerprint is instead a continuous (amplitude modulated) signal along the DNA and includes DNA melting maps [36, 37], DNA competitive binding maps [28, 38] and dense enzymatic labeling of DNA [39]. In the present study, DNA molecules are labeled using the competitive binding assay (dense labeling).

Competitive binding optical maps are created by introducing the DNA into a mixture of YOYO-1 and netropsin. Netropsin (non fluorescent) binds to DNA preferentially in AT-rich regions, while YOYO-1 (fluorescent) will bind in the regions that are left (GC-rich) since it is not sequence specific. As a consequence, GC-rich sequences are observed brighter than AT-rich sequences. The result is an alternating pattern of dark and bright regions which is a fingerprint, or barcode, of the DNA molecule. The DNA used in this article is plasmids from bacteria, which are circular DNA molecules separated from the chromosomal DNA, that can store, for instance the genes responsible for antibiotic resistance.

The stretching of DNA molecules in optical DNA mapping assays is achieved by surface adsorption or using nano-channels, where each of the two methods has its pros and cons. For instance, by using surface adsorption the DNA is perfectly still during imaging, but non-uniform stretching may occur, which introduces challenges for creating reproducible barcodes. Even though optical maps of surface adsorbed DNA has successfully used in high throughput applications [40], nanochannels are expected to be better suited for high throughput purposes, as an arbitrary number of molecules can be, essentially continuously, run through the experimental array and analyzed [1]. The main post-processing challenge for nanochannel-based methods is that the stretched DNA molecules are not completely still; thermal center-of-mass motion and conformational fluctuations still take place. These local and global longitudinal movements of the DNA introduces challenges for how to calculate reproducible time-averages of long measurements (typically, a few hundred time frames).

**Fig 1:**
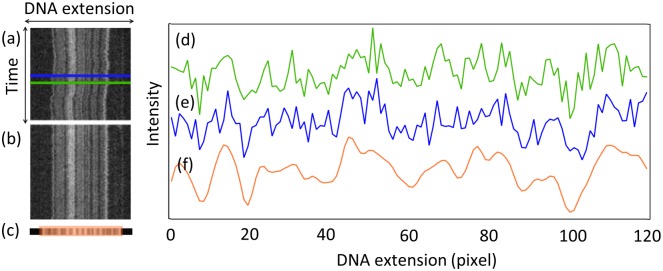
Examples of optical mapping kymographs (raw, aligned and time averaged) from a linearized plasmid DNA stretched in a nanochannel. (a) A raw kymograph (i.e. a stack of images of stretched and fluorescently labeled DNA) from a linearized plasmid obtained using the competitive binding assay described in [38]. The horizontal direction corresponds to the nano-channel extension (i.e., the direction of the stretched DNA) and vertical axes are different time points (0.1 s between time frames). The kymograph consists of 200 single time frame images (d, e). (b) The raw kymograph is aligned (using *WPAlign* from [41]) and subsequently averaged over all 200 time frames in order to produce (c, f) noise-reduced, time averaged DNA barcodes. The noisy curves in (d, e) represent the intensities along two single time-frame (snap-shot) barcodes, see (a). For visualization purposes, the snap-shot barcodes were shifted globally to the position where they have the maximum correlation coefficient with the time-averaged barcode. The challenge addressed in this study is how to make the noisy single-time frame barcodes of the form illustrated above resemble the (more reproducible) time-averaged barcode to a higher degree by using low-pass filtering. The barcodes shown are from plasmid *pEC005A*, see [24] for further information about the experiments.

The currently most effective method for creating reproducible DNA barcodes from nano-channel based experiments is to use the time average of an *aligned kymograph*. A kymograph is a stack of, typically, a few hundreds of time-frames taken one after another (Fig 1a). Once such a raw kymograph has been obtained, the different time frames are aligned to each other using global shifting and local stretching [37, 41], resulting in an aligned kymograph (Fig 1b). Finally, an average over the time frames of the aligned kymograph produces a time-averaged DNA barcode (Fig 1c and 1f), which has much less noise than any single time frame barcode (Fig 1d and 1e). Recent progress allows for reasonably efficient kymograph alignment using the WPAlign method where the computational time scales linearly with the number of pixels in the barcode [41]. For the plasmids used in this study, kymograph alignment using WPAlign takes a few minutes on a modern laptop computer, whereas for a whole human genome the alignment would take about an hour. These computational times may be prohibitive for high throughput measurements where thousands, or millions, of optical maps need to be aligned and averaged.

As an alternative to the strategy of recording movies of nano-channel stretched DNA (including subsequent alignment and time-averaging of kymographs as described above), we herein describe a new post-processing method for reducing the random noise in a *single time frame* DNA barcode (measured during 0.1s). The introduced methodology will allow experimentalists to turn single “snap-shot data” [39], into barcodes which are more similar to the time average of aligned kymographs. Thus, our method tackles the problem of how to make a single time frame barcode best “mimic” a barcode which would be obtained from a perfectly still, fluorescently labeled DNA molecule from which an infinite number of photons were collected. The method introduced is based on applying a low-pass filter on single time frame barcodes. In practice, we apply the filter by iteratively increasing the “strength” of the filtering, until all peaks have a spatial width of at least the width of the point spread function of the system (a known parameter). The use of filters for noise reduction on images has been studied numerous times [42–50], but such methods often require a calibration to find the best value for the parameters of the filter. Some other studies also aim at finding the optimal parameter, using only information from the image [51–54]. However, this study is the first to introduce a filtering method for snap-shot DNA barcodes and that is parameter free. Although the method developed herein is applied to competitive binding barcodes, our method is likely to apply essentially “as is” to all barcodes of type (ii), see above. We do not expect our method to be of use for restriction enzyme cut DNA fragmentation maps [32, 33]. On the other hand, for the case of sparse enzymatic labeling [35, 55] we believe that our method might also be effective for making a snap shot experiment better mimic the perfect time-average scenario (infinite number of photons). Possibly, however, minor modifications could be required for these barcode types.

## Methods

Our method for reducing the random noise in a single time-frame DNA barcode is a post-processing method based on the application of a low-pass filter. In this section we describe and justify our method. We describe the data sets and the approach we use for validation. We also describe our approach for testing our method’s plasmid identification (ID) capabilities (matching filtered single time frame barcodes to the correct plasmid from a database of plasmid theory barcodes).

### Filtering method

Low-pass filters are commonly used in signal processing for reducing high-frequency noise. Examples of low-pass filters are the Window-Sinc, Moving Average and Gaussian filters. A brief description about these types of filter can be found in Section. Each filter has its own pros and cons but all of them require an input parameter that determines to which degree the signal, or barcode, is filtered, i.e. its filtering power. For instance, the Window-Sinc filter requires a cut-off frequency *f*_cut-off_, the Moving Average filter needs a certain neighborhood for averaging *b*, and the Gaussian filter a standard deviation *σ*. The optimal choice for the parameters depends on the properties of the recorded signal, but is in general not immediately clear how to choose these parameter values without trial-and-error and visual inspection of the filtered signal. In order to circumvent the arbitrariness in the choice of the filtering parameter, we here base our approach on a measurable parameter, namely the FWHM (Full Width at Half Maximum) of the Point Spread Function (PSF) of the system. We here use the FWHM as the unique parameter in the filtering process by applying the low-pass filter to the single time-frame recursively, increasing its filtering power until all peaks become at least as wide as the FWHM of the PSF. The rationale behind this method is that the fluorescence from a single fluorescent point source is well-described by a Gaussian function with a standard deviation *σ*_psf_. If the point source is kept perfectly still and an infinite number of photons are recorded (i.e., a perfect time-average) then this Gaussian is perfectly smooth and has a FWHM given by

$$\begin{array}{c} \omega_{\text{psf}}=2.355\sigma_{\text{psf}} \end{array}$$ (1)

In our case *σ*_psf_ ≈ 300 nm [28] and hence *ω*_psf_ ≈ 707 nm ≈ 4.5 pixels (one pixel is 159 nm in the experiments analyzed herein). We elaborate further on the point spread function at the end of this section. Based on the reasoning above, we note that a perfect time-average should have no peaks with FWHM smaller than *ω*_psf_, which justifies our recursive filtering method as detailed below. In short, our method is:

1. Determine *σ*_psf_ for the experimental setup and set a threshold value, *ω*_thresh_, equal to the associated FWHM of the PSF, see Eq (1), i.e., set *ω*_thresh_ = *ω*_psf_.
2. Set the filter parameter (*f*_cut-off_, *b* or *σ*) to its start value. The start value is chosen to correspond to essentially no filtering: for the Window-Sinc filter *f*_cut-off_ is set to the largest allowed frequency (Nyquist frequency), for the Moving average *b* is set to 1 pixel and for the Gaussian filter we set *σ* to 1 pixel.
3. Apply the low-pass filter with the given value of the filter parameter.
4. Detect all peaks in the barcode and measure the width of each of them. The specific method we use for measuring the widths is discussed in S1 Fig.
5. Check if the FWHM (*ω*_peak,*k*_) of each peak *k* in the filtered barcode is greater than the threshold *ω*_thresh_. If indeed *ω*_peak,*k*_ ≥ *ω*_thresh_ for all *k*, then we stop the algorithm. If this is not the case, update the filter parameter (decrease *f*_cut-off_, increase *b*, or increase *σ*, respectively) and go back to step 3.

Let us make a few comments on our algorithm. First, we note that in our recursive method, the optimal parameters (e.g. *f*_cut-off_, *b* or *σ*) are determined indirectly by setting the minimum FWHM that all peaks in the filtered barcode must have. Second, our method is not limited to the three filters presented here but any other low-pass filter can be used provided that its filtering power parameter can be controlled and increased gradually in each iteration. Third, a method for estimating the widths *ω*_peak,*k*_ of the, often overlapping, peaks is needed in step 4 of our method. In S1 Fig we explain the algorithm we used for that purpose. Fourth, a perfect Gaussian form of the PSF (the expected emission pattern of a point source) does not necessarily apply to our data. For instance, during the 0.1 s of imaging for a single time frame each fluorescent molecule along the DNA will possibly move slightly due to center-of-mass motion and local conformational DNA fluctuations, thereby causing motional blurring. Moreover, the kymograph alignment procedure used here [41] is not perfect, which causes additional broadening of the peaks in time-averaged barcodes. The value used here, *σ*_psf_ = 300 nm, however, appears to well incorporate all these effects, as indicated by the good agreement between theory barcodes (calculated using *σ*_psf_ = 300 nm) and experiments [24]. Lastly, above we approximated the PSF by a Gaussian with standard deviation *σ*_psf_. For such a function, Eq (1) relates the FWHM to the standard deviation of the PSF. However, high numerical aperture objectives could require a more complex expression for the PSF [56, 57]. In such a case our method still applies, but one must then use the known FWHM of this new PSF in our algorithm above.

### Mathematical expressions for the filters used

Let us now describe mathematically the three low-pass filters which were introduced in Sec. Filtering method. Denote by ***x*** a vector with measured (noisy) intensity values (bold symbols denote vectors and non-bold symbols denote the components of a vector). We assume that for each pixel *j* = 1, …, *N*, the measured intensities (the components of ***x***) can be written *x*(*j*) = *y*(*j*) + *n*_*y*_(*j*) + *n*_bg_(*j*), where ***n***_bg_ is a zero mean random noise vector (additive noise), ***n***_*y*_ is signal-dependent noise, and ***y*** is the noise-free DNA barcode (perfect time average). The shot noise, ***n***_***y***_, includes fluctuations due to the Poisson distribution of photons and electronic read-out effects, whereas ***n***_bg_ is background noise which is independent of ***y***. The shot noise is a Poisson distributed random variable [58] in general. Using the Gaussian approximation of the Poisson distribution (valid when the number of collected photons ≫ 1) we have that $n_{y}\left(j\right)=\eta \left(0,1\right)\sqrt{y(j)}$, where *η*(0, 1) is a Gaussian random variable with mean 0 and standard deviation 1. In the equations above, we neglected blurring effects due differences in a DNA molecule’s lengths at two different times due to conformational fluctuations (these effect are reduced by stretching single-time frame barcodes to the same length as time-averaged barcodes, see Sec. Validation method).

The idea of applying a filter to the measured signal, ***x***, is to make the filtered signal, here denoted by $\tilde{y}$, more closely resemble the perfect time average ***y***. We here only use linear filters, which mathematically are expressed as:

$$\begin{array}{c} y\left(j\right)\approx \tilde{y}\left(j\right)=(h*x)\left(j\right)=\sum_{k=1}^{N}h\left(j-k\right)\;x\left(k\right),\;j=1,...,N. \end{array}$$

By the convolution theorem, in frequency domain this expression transforms to

$$\begin{array}{c} \tilde{Y}\left(f_{n}\right)=H\left(f_{n}\right)\;X\left(f_{n}\right),\;f_{n}=\frac{n}{N}\cdot {\text{pixel}}^{-1},\;n=-\frac{N}{2},...,0,...,\frac{N}{2}-1, \end{array}$$

where ***H***, ***X***, $\tilde{Y}$ and ***Y*** are the Fourier transforms of ***h***, ***x***, $\tilde{y}$ and ***y***, respectively, and ***f*** is a vector with allowed frequencies.

From the nature of the noisy (single time frame barcode) and noiseless (time averaged barcode) signals, see Fig 1, we observe that in order to improve the resemblance of the single time frame barcode to the time-averaged barcode, we must choose ***H*** to be a low-pass filter, i.e. the filter must act to reduce or eliminate the highest frequency components in ***x***. Information about the three filters used in this study can be found in Table 1.

**Table 1:** Summary of three low-pass filters used in this study: Gaussian, Moving average and Window-Sinc filter.

Filter	Gaussian	Moving average	Window-Sinc
Description	Assigns to each pixel the weighted average of all its neighbors, with weights defined by a Gaussian density function (standard deviation *σ*) centered at that pixel.	Assigns to each position the average of the intensity over its *b* nearest neighbors (included the position itself). By symmetry *b* is odd.	Sets to zero all the components of frequency higher than *f*_cut-off_. Filtering function is a box-car function in frequency domain and a Sinc function in space domain.
Math. expr.	$\tilde{y}\left(j\right)=\frac{\sum_{k=1}^{N}\;x\left(k\right)\exp(-{\left(k-j\right)}^{2}/\left(2\sigma^{2}\right))}{\sum_{k=1}^{N}\exp(-{\left(k-j\right)}^{2}/\left(2\sigma^{2}\right))}$	$\tilde{y}\left(j\right)=\sum_{k=-(b-1)/2}^{(b-1)/2}\frac{x(j+k)}{b}$	*H*[*f*_*n*_] = 1 if |*f*_*n*_| ≤ *f*_cut-off_ and *H*[*f*_*n*_] = 0 if |*f*_*n*_| > *f*_cut-off_
Parameter	Standard deviation, *σ*	Window size, *b*	Maximum frequency, *f*_cut-off_

### Validation data set

We test our filtering method for reducing noise on single frame barcodes on the experimental data from [24] (DNA barcodes of bacterial plasmids obtained from competitive binding assays). The data set consists of 32 kymographs with a total 6400 single time frame barcodes. Eight of the plasmid molecules are type *pUUH* (barcodes of length 360 ± 23 pixels), eleven *pEC005A* (barcodes 127 ± 7 pixels long) and thirteen *pEC005B* (barcodes 247 ± 15 pixels long). Each single frame consists of 512 pixels, with the barcode signal in the center, and approximately the first and the last hundred pixels are the background (the number of pixels corresponding to the background depends on the length of the molecule measured). To extract the central region we use the Otsu method [59] to choose a threshold intensity to discriminate background (‘0’) from signal (‘1’). Using this threshold we detect the edges of the signal region, as points where the signal makes ‘0’-‘1’ and ‘1’-‘0’ jumps, respectively. The intensity threshold in the Otsu method is determined by minimization of the intraclass variance of the intensity histograms (we use the histogram of the complete kymograph instead of the single frames for more robust values of the threshold). Some characteristic features of the experimental data set are summarized in S1 Table. More information about the experiment and the plasmids can be found in [24]. We apply the three filters specified in Table 1 to each of the 6400 single time frames in this data set.

### Validation method

For validating that our filtering method indeed improves the resemblance of a filtered single time frame (*fst*) barcode to the associated time average of its kymograph (*ta*), we compare the Pearson correlation coefficient $\hat{C}$ between the single frame barcode and *ta* before and after applying the filter. The Pearson correlation coefficient between two vectors ***x*** and ***y*** is defined in Eq (2).

$$\begin{array}{c} {\hat{C}}_{x,y}=\frac{1}{N-1}\frac{\sum_{j=1}^{N}(x\left(d+j\right)-\mu_{x})(y\left(j\right)-\mu_{y})}{\sigma_{x}\sigma_{y}} \end{array}$$ (2)

where ***x*** and ***y*** are the two intensity vectors being compared (the single time-frame barcode and the kymograph time average), *N* is the length of the barcodes in pixels, *μ*_***x***_ and *μ*_***y***_ are the mean value of ***x*** and ***y*** respectively, and *σ*_***x***_ and *σ*_***y***_ their standard deviations. As in [24] we use bit-weighting to mask the ends of the barcode, i.e. end pixels within a distance 3*σ*_psf_ from the edges are not included when calculating the correlation coefficient above. ${\hat{C}}_{x,y}$ takes values between -1 and 1, taking value 1 when the barcodes are identical, and 0 when they are uncorrelated. The quantity *d* is a relative global shift between the two barcodes ***x*** and ***y*** (a few pixels). This quantity is used herein since due to center-of-mass diffusion two different time frames will in general slightly displaced with respect to each other. Also, before comparing the two barcodes, we stretch/shrink the single time frame to the same length as the time average (using linear interpolation) to compensate for fluctuations in DNA barcode length between time frames. In the rest of the text, results for the Pearson correlation coefficient are at optimal shifts *d* (i.e., $\hat{C}$ is calculated as in Eq (2), where *d* is the position that maximizes $\hat{C}$) and with single time frames stretched to the same lengths as the time-averaged barcode.

### Comparing experimental barcodes to database of plasmid theory barcodes

To address the question whether filtered barcodes are useful in plasmid “fingerprinting” applications we used the 3127 plasmid theory barcodes from [24]. The aim is to match an experimental plasmid barcode to this database and, ideally, identify the correct plasmid. To that end, we match experimental barcodes to the theory database and calculate $\hat{C}$ values. We only match to theory barcodes which have similar lengths as the experimental barcode (here, defined as a length within ±3*σ*_length_, where *σ*_length_ is the standard deviation in the lengths of the experimental single time-frames). For every experimental barcode, we thus get a set of $\hat{C}$ values. We then turn this set into a histogram and fit to a Gumbel probability density, $\phi (\hat{C})$ [24]. Based on $\phi (\hat{C})$ we define a measure, the separability score (s-score), s-score = $\int_{{\hat{C}}_{\text{observed}}}^{\infty }\phi \left({\hat{C}}^{\prime }\right)d{\hat{C}}^{\prime }$, which quantifies how far out in the tail the observed correlation coefficient, ${\hat{C}}_{\text{observed}}$ (coefficient obtained by matching the experimental barcode to the “true” plasmid theory barcode), is. In [24] an analytical expression for the s-score is provided. The s-score is, by construction, in the range [0, 1], and an s-score close to 0 means that the correct plasmid has a correlation coefficient far out in the tail of the histogram, and is well separated from the rest. In contrast, an s-score around 0.5 corresponds to bad separability. Note that the s-score is defined similarly to a p-value [24], and hence the s-score is an approximation to the expected number of false positive when matching the experiment to the database. As ‘experimental barcodes’ used for matching to the database we consider: (*ust*) unfiltered single time-frame barcodes, (*fst*) filtered single-time frame barcodes and (*ta*) time-averaged barcodes.

## Results

**Fig 2:**
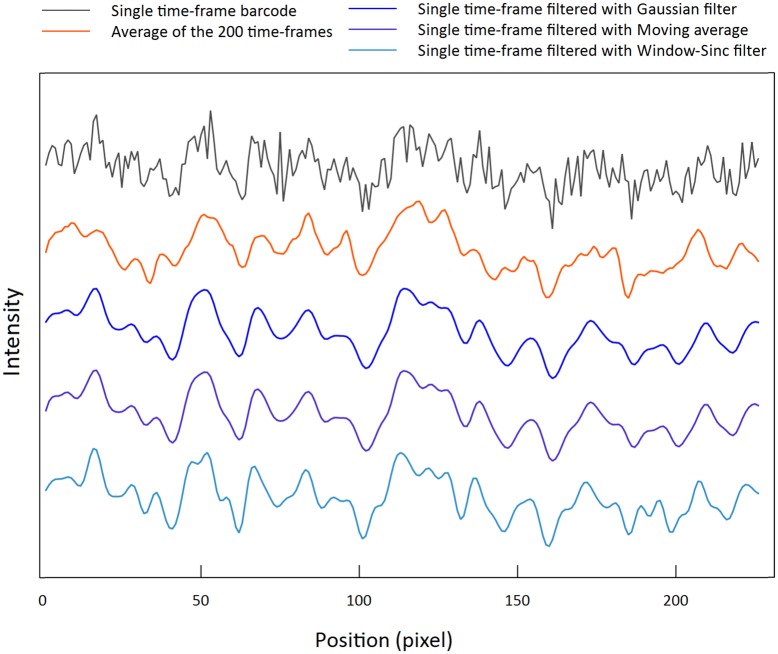
Example barcode filtered using our noise-reducing filtering method. (grey) A noisy single time-frame (snap-shot) barcode taken with 0.1s exposure time. (orange) The time average of the aligned kymograph. Such time-averages are used as reference (“true” barcode) throughout this study and used to judge the quality of the filtering process. (blue barcodes) From top to bottom Gaussian, Moving average and Sinc filter, respectively, is applied recursively to the single-time frame barcode (grey) until all peaks in the filtered barcode have a FWHM of at least that of the FWHM of the PSF of the system. Notice the visual similarity of the filtered barcodes to the time-averaged barcode. The Pearson correlation coefficient between the time average of the aligned kymograph and the barcode before and after the the filtering changes from 0.6 without the filtering, to 0.8 after filtering. The original raw kymograph consists of 200 single time-frame shots (exposure time 0.1s) from plasmid *pEC005B*.

In practice, single time-frame barcodes filtered with our method show a great degree of visual similarity to the associated time-averaged barcodes, see Fig 2 (*pEC005B* plasmid), where the three filters from Table 1 were applied to a single-time frame barcode. More examples can be found in S2 and S3 Figs. For the example in Fig 2 the Pearson correlation coefficient between the time-averaged barcode and the filtered single time frame barcode yields a value ≈ 0.8 for all the three filters. In contrast, the unfiltered and the time-averaged barcodes have a correlation coefficient of only ≈ 0.6. Thus, for this particular example, our method succeeds very well at making the filtered barcode better mimic the time-averaged barcode (33% improvement in the correlation coefficient).

**Fig 3:**
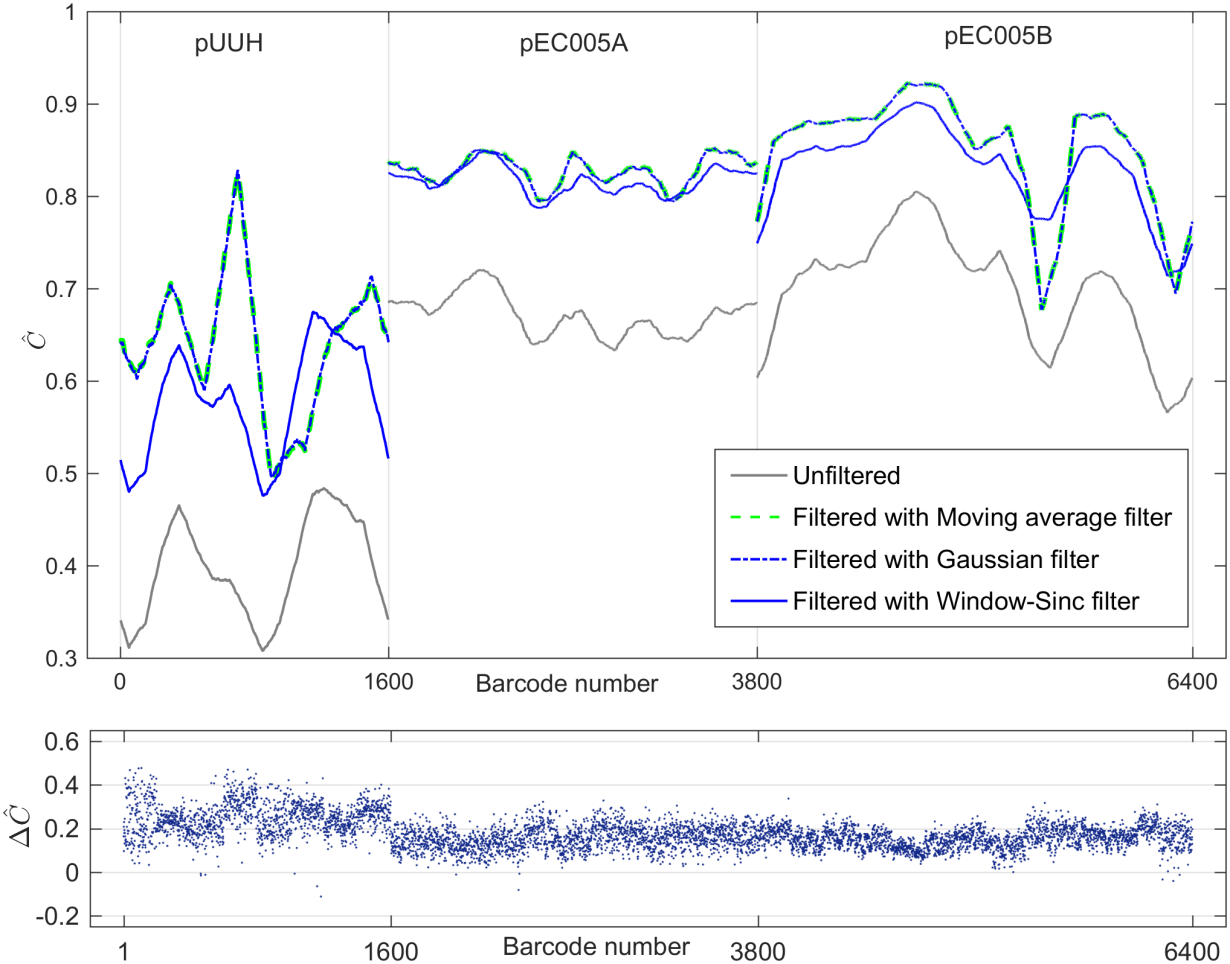
Change in the Pearson correlation coefficient between each single time-frame barcode and its aligned kymograph time average after application of the low-pass filter. The first 1600 barcodes originate from the *pUUH* plasmid, barcodes 1601-3800 are from plasmids *pEC005A*, and barcodes number 3801-6400 from plasmids (*pEC005B*). In grey is shown the correlation coefficients for the case without filtering, and in blue/green the correlation after filtering. The four lines in the top plot are smoothed for visualization purposes and are moving averages of the result over the 300 nearest neighbor barcodes (each of the three plasmid types treated separately). The bottom panel shows the change $\Delta \hat{C}={\hat{C}}_{fst,ta}-{\hat{C}}_{ust,ta}$, for all 6400 single time-frames for the case of Gaussian filter. Here, ${\hat{C}}_{fst,ta}$ is the correlation coefficient between the filtered single time-frame (*fst*) barcode and the time averaged (*ta*) barcode, and ${\hat{C}}_{ust,ta}$ is the correlation coefficient between the unfiltered (noisy) single time frame barcode and the kymograph time average. Notice the rather dramatic improvement in correlation with the time-averaged (“true”) barcodes after filtering. In all of the cases the filter parameter used was the (known) FWHM of the PSF of the system *ω*_psf_. Here, *ω*_psf_ = 4.5 pixels.

In order to further quantify the apparent visual similarity between filtered and time-averaged barcodes, we applied our method to all 6400 single time-frame barcodes from the 32 kymographs (200 time frames each), see Sec. Validation data set. We find that the findings from Fig 2 hold generally, as illustrated in Fig 3, which shows the Pearson correlation coefficient (Eq (2)) between all 6400 single time frame barcodes, using filtering and no filtering, and the associated time-averaged barcodes. It is apparent that filtering does indeed significantly improve the agreement between single time frame barcodes and the true time-averaged barcode. As a minor remark, we notice (see S1 Table) that the mean intensity after background intensity subtraction (this quantity is proportional to the number of collected photons from the fluorescent labels on the DNA) is more than a factor 2 smaller for *pUUH* compared to *pEC005A* and *pEC005B*. The shot noise is hence more pronounced for the pUUH experiment, which is likely one of the major reasons that (see Fig 3) the *pUUH* molecules (first 1600 time frame barcodes) have an approximately 0.2 points lower original correlation than the *pEC005A* and *pEC005B* plasmids (time frame barcodes 1601-6400).

By averaging the results in Fig 3 over all barcodes and over the three filters we get a single number quantifying the effectiveness of our method, namely, the average change in correlation coefficient, $\Delta \hat{C}$, after and before filtering (Table 2). We find that the average value for $\Delta \hat{C}$ is 0.17 ± 0.06 points. It is noteworthy that the effectiveness of the three filter types is very similar. In S4 Fig we show results for $\Delta \hat{C}$ obtained by varying *ω*_thresh_. For the choice *σ*_psf_ = 300 nm used here, we find that indeed the best improvement in correlation coefficient occurs close to *ω*_thresh_ ≈ *ω*_psf_ for the Gaussian and moving average filters (for the Window-Sinc filter the optimal is slightly shifted to *ω*_thresh_ ≈ 1.5*ω*_psf_). As argued in Sec. Filtering method, the choice *σ*_psf_ = 300 nm is a rough estimate, which includes motional broadening effects during exposure, effects due to imperfect kymograph alignment etc. Nevertheless, setting *ω*_thresh_ = *ω*_psf_ = 300 nm seems to be close to optimal.

**Table 2:** Increase in the Pearson correlation coefficient between filtered a single time frame and the aligned kymograph time average. The table shows the improvement, $\Delta \hat{C}$, in the Pearson correlation coefficient, $\hat{C}$ (see Eq (2)), between each single time frame barcode and its aligned kymograph time average after filtering the single frame. Correlation coefficients were averaged over all 6400 time-frame barcodes (see Fig 3) for each type of filter used (Gaussian, Moving Average and Windowed-Sinc filter). The improvement is defined as $\Delta \hat{C}={\hat{C}}_{fst,ta}-{\hat{C}}_{ust,ta}$, where ${\hat{C}}_{fst,ta}$ is the correlation coefficient between the filtered single time-frame (*fst*) barcode and the time averaged (*ta*) barcode, and ${\hat{C}}_{ust,ta}$ is the correlation coefficient between the unfiltered (noisy) single time frame barcode and the kymograph time average. We see that all filters lead to a similar average improvement in the correlation, roughly 0.2 points. Results for $\Delta \hat{C}$ by type of plasmid (*pUUH*, *pEC005A*, *pEC005B*) are found in S1 Table in Supplementary Information.

Average improvement in $\hat{C}$ over all barcodes		
Type of filter	$\langle \Delta \hat{C}\rangle =\langle {\hat{C}}_{fst,ta}-{\hat{C}}_{ust,ta}\rangle$	$\sigma_{{\hat{C}}_{fst,ta}-{\hat{C}}_{ust,ta}}$
Gaussian	0.18	0.07
Moving Average	0.18	0.07
Windowed-Sinc	0.16	0.05
Mean over all types	0.17	0.06

The final (optimal) values of the filter parameters after recursive denoising are shown in S5 Fig, where we also list rough suggested values for these parameters. Note, however, that the rough suggestions must be used with care as they may be specific to the present data set (based on the competitive binding assay). In contrast, we expect the general parameter-free method introduced herein to be applicable to most types of optical maps.

**Fig 4:**
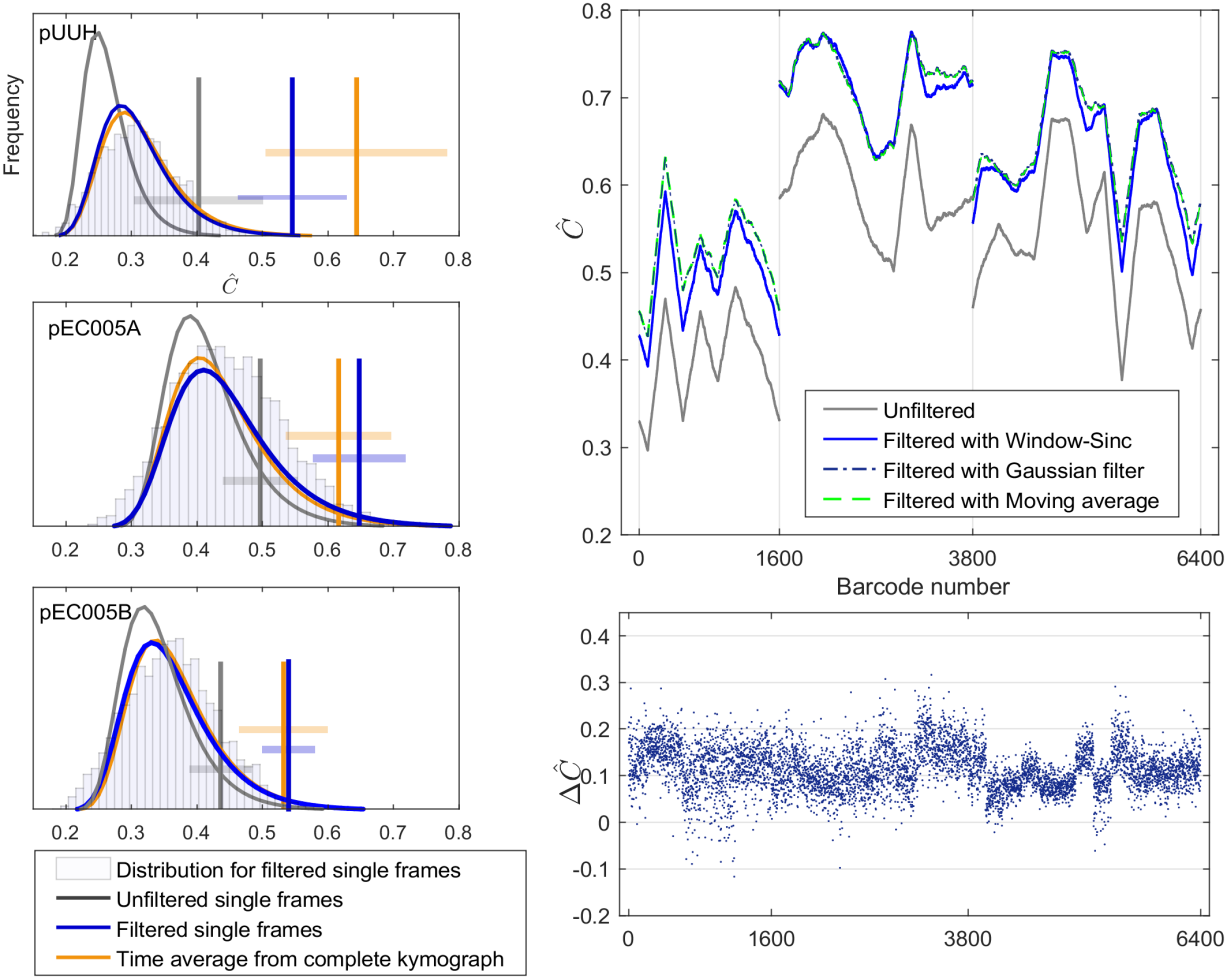
Plasmid ID by comparing individual experimental barcodes to a theory database. (Left) Pearson correlation coefficients between the experimental barcodes (*pUUH*, *pEC005A* and *pEC005B*) and the plasmid theory database (as in [24]) were calculated and turned into histograms. As input experimental barcode we used: unfiltered single-time frame (*ust*) barcodes, filtered (with Gaussian filter) single-time frame (*fst*) barcodes, and time-averaged (*ta*) barcodes. The vertical line gives the correlation coefficient for the correct plasmid. Notice that *ust* barcodes are not very good at plasmid ID (s-scores between 0.4-10%), but, *fsb* and *ta* barcodes are better (with s-scores less than 3%). Experiments were only matched to theory barcodes which had a length within ±3*σ*_length_ of the experimental barcode, with *σ*_length_ = {23, 7, 15} pixels for *pUUH, pEC005A and pEC005B*, respectively. The Gumbel fits to the histograms were done using the same method as in [24]. (Right) The panels on the right show the change in $\hat{C}$ between the experiment and the theoretical barcode (i.e., theory barcodes from *pUUH*, *pEC005A* or *pEC005B*, respectively) after filtering single time frame barcodes. On the top-right plot, in grey is shown the correlation coefficients for the case without filtering, and in blue/green the correlation after filtering. The four lines in the top plot are smoothed for visualization purposes and are moving averages of the result over the 300 nearest neighbor barcodes (each of the three plasmid types treated separately). The right-bottom panel shows the change $\Delta \hat{C}={\hat{C}}_{\text{filtered}}-{\hat{C}}_{\text{unfiltered}}$ for all 6400 single time-frames for the case of Gaussian filter. On average, the single frames match to theory improves by 0.11 ± 0.04 points after filtering (average over all three filters).

Let us now investigate whether filtered single-time frame (*fst*) barcodes are useful in plasmid ID applications. To address this question we match experimental barcodes to the plasmid database and compute $\hat{C}$ and s-scores, see Sec. Comparing experimental barcodes to database of plasmid theory barcodes. The results are found in Fig 4 which show $\hat{C}$-histograms for *fst* (filtered single time-frame using the Gaussian filter) barcodes along with the correlation coefficients for the correct plasmid (vertical lines). Shown are also Gumbel fits, where for *ust* (unfiltered single time-frame) and *ta* (time averaged) barcodes we show only Gumbel fits for visual clarity. We find that that there are {320, 428, 300} theory barcodes which are similar in length (within 3 standard deviations in length compared to experiments, see Methods) for *pUUH*, *pEC005A* and *pEC005B*, respectively. For *ust* barcodes, the correlation coefficient for the correct plasmid is not always well-separated from the rest (s-scores = {0.0044, 0.10, 0.069}, which were obtained by converting the average $\hat{C}$ to a separability score, for the plasmids *pUUH*, *pEC005A* and *pEC005B*). Thus, *ust* barcodes cannot, for the plasmids considered here, reliably be used in plasmid ID applications. In contrast, *fst* barcodes are better at plasmid ID: visually the correlation coefficient for the correct plasmid ends up further out in the tail of the histogram and the associated s-scores = {0.0020, 0.027, 0.021} are indeed lower (expected fraction of false positives are thus 0.2%, 3% and 2% for *pUUH*, *pEC005A* and *pEC005B*, respectively). In fact, these s-scores are comparable to the s-scores for time-averaged barcodes ({5.4 × 10^−4^, 0.030, 0.026}).

## Summary and discussion

We designed a post-processing method for reducing the random noise in single time frame optical DNA maps (DNA barcodes). Our method consists of applying a low-pass filter recursively until all features in the filtered signal have a width of at least the FWHM (Full Width at Half Maximum) of the optical PSF (Point Spread Function) of the system. For testing the method, we used 32 kymographs of 200 time frame barcodes each (in total 6400 noisy time frame barcodes). For quantifying the quality of the filtering, we compared each single noisy barcode and its filtered counterpart to the corresponding aligned kymograph time average (“noiseless”, or “true”, barcode). We used the Pearson correlation coefficient to quantify the similarity between barcodes, and we find that filtering improves the correlation coefficient by 0.17 ± 0.06 points (comparing single frames to the kymograph time average) and 0.11 ± 0.04 (comparing single frames the barcode from the theory, Fig 4, right) on average for the three different filters tested (Gaussian, Moving Average and Windowed-Sinc filter). The three low-pass filters display comparable effectiveness. We have here used three particular filters but the methodology can be applicable to other type of low-pass filters provided that one knows how to recursively increase the filtering power of the chosen filter.

Another commonly used filter for noise reduction is the Wiener filter (and different variants thereof) [60]. The Wiener filter achieves minimum squared error for additive, signal-independent noise and uses the spectral density of the background noise as input. In our case, there are *two* sources of noise. The background noise which is indeed additive and signal independent, and the shot noise, which is due to fluctuations in photon count (for finite number of photons there are deviations from the recorded signal and a perfectly smooth Gaussian) and is signal dependent. One of the conditions for the Wiener filter to achieve a least square error solution is that noise and signal have to be uncorrelated, which is not the case for shot noise. For that reason, we do not recommend the standard Wiener filter for noise reduction in single time frame DNA barcodes of the type considered here where shot noise effects are prominent. Possibly, the *Adaptative* Wiener filter [61] which uses the input parameter *neighborhood of influence* of a pixel, can be used within our framework.

The increase in Pearson correlation coefficient using our method is rather significant but we do not reach a perfect match (correlation coefficient = 1) between time averaged barcodes and filtered barcodes. What fundamental limitations does our method have? First, we note that the DNA extension is a fluctuating quantity [62], i.e., different single time frame DNA barcodes will by necessity be of slightly different length. This effect will provide non-perfect similarity between filtered and time-averaged barcodes (we use linear interpolation to stretch single time frame barcodes to the same length as the time-average barcode). Second, local stochasticity is added to the single time frame DNA barcode since during the 0.1 s of imaging of a single frame, there will be minor local DNA fluctuations leading to random motion of the fluorescent molecules attached to it. Third, the kymograph alignment algorithm, WPAlign [41], does not produce perfectly aligned kymographs. Thus the time-averages (here used as “true” barcodes) contain deviations from the hypothetical scenario that the DNA was perfectly still and imaged for an infinitely long time. Fourth, noise fluctuations are often well described by “white” noise, i.e., all noise frequency components have equal weights. Thus, even if high frequency components are removed (as is done here), the final filtered barcode will always contain the low frequency components of the noise.

Our analysis further shows that filtered single time frame barcodes can be used for plasmid ID applications. We find that the expected fraction of false positive matches is less than 3% when matching filtered single time-frame experimental barcodes to a database of plasmid theory barcodes. This is a significant improvement compared to using unfiltered barcodes.

The results in this study originate from DNA barcodes from nano-channel based competitive binding assays. However, the method might also to be effective on single time frame DNA barcodes from other types of optical DNA mapping experiments, including barcodes with sparse enzymatic labels.

Finally, we point out that the filtering process takes less than 0.5 s per barcode on a standard laptop computer. Due to the speed with which experimental snap-shot data can be recorded (a single time frame is 0.1 s) and the real time character of our filtering methods (fraction of a second), we believe that the method we present herein will prove useful in high-throughput optical mapping applications.

## Supporting information

S1 Table Description of the experimental DNA barcode data. From left to right: *I*_*s*_ = mean intensity of the signal part of the barcodes (a.u.), where the errors are the standard deviation over different molecules, *σ*_*s*_ = the standard deviation in intensity for single time frame barcodes around the time-averaged barcode (a.u.), *I*_bg_ = mean intensity level of the background (a.u), *σ*_bg_ = standard deviation in the background intensity (a.u), length = average lengths of molecules (pixels), *σ*_length_ = average of standard deviations in length in a kymograph (pixels). Brackets, 〈…〉, corresponds to an average over different molecules.
(PDF)

S2 TableIncrease in Pearson correlation coefficient $\hat{C}$ by type of barcode after applying our method.Change in $\hat{C}$ between the single time-frame and the aligned kymograph time average after reducing the noise with our method with the three filters (Gaussian, Moving average and Window-Sinc) independently. The values are an average over the three filters and over all time-frame barcodes of the same type (*pUUH*, *pEC005A* and *pEC005B*). ${\hat{C}}_{ust,ta}$ is the correlation between the unfiltered single time-frame (*ust*) barcode before any filtering and its aligned kymograph time average (*ta*). ${\hat{C}}_{fst,ta}$ is the correlation between the filtered barcode (*fst*) and the aligned kymograph time average. We see that in *pUUH* barcodes (the longest DNAs) the correlation improves slightly more than for the other two barcode types (*pEC005A* and *pEC005B*).(PDF)

S1 Fig Estimated full width at half maximum (*ω*) of a peak in overlapping peaks. For estimating *ω* when peaks overlap, which is the case in our densely labeled barcodes, we use an algorithm implemented in Matlab Version 2015b in the function *findpeaks(PeakSig, x, ‘WidthReference’, ‘halfheight’)*, where *PeakSig* is the intensity signal, *x* the position (pixels in our case) and *‘WidthReference’, ‘halfheight’* indicates the method for estimating the width of the peaks. In short, this function finds all maxima and estimates the full width at half maximum as follows: 1) Find all local maxima (peaks) (blue triangles). 2) The height of a peak is defined as the vertical distance between its maximum value and 0 (light blue line). 3) Detect the local minima on both sides of the peak. If a local minimum is not of intensity 0, draw a vertical line from that local minimum to 0 (blue line). 4) The estimated FWHM, *ω*, is then the distance, measured at half height of the peak, between the peak signal, or the drawn vertical line, from one side of the maximum of the peak to the other (red line). As shown in the examples in the figure, this method is rather successful at estimating the “true” width, i.e. 2.355*σ*, in a scenario of overlapping Gaussians.
(TIF)

S2 Fig Noisy barcode from a plasmid *pEC005B* filtered using our method. (a) Time average of the aligned kymograph (“noiseless” barcode). (b) Frame 100 (measured during 0.1s) extracted from the kymograph (noisy barcode). The three barcodes (c)-(e) are the result of using our method to reduce the noise with: (c) a Gaussian filter, (d) a Moving average filter, (e) a Window-Sinc filter. The Pearson correlation coefficient between the time average of the aligned kymograph and the single frame barcode improves by ≈0.15 points after filtering, with any of the three filters (from 0.75 before filtering, to ≈0.9 afterwards).
(TIF)

S3 Fig Noisy barcode from a plasmid *pUUH* filtered using our method. (a) Time average of the aligned kymograph (“noiseless” barcode). (b) Frame 100 (measured during 0.1s) extracted from the kymograph (noisy barcode). The barcodes (c)-(e) are the result of using our method to reduce the noise with: (c) a Gaussian filter, (d) Moving average filter, (e) a Window-Sinc filter. The Pearson correlation coefficient between the time average of the aligned kymograph and the single frame barcode improves in ≈0.2 points after filtering, with any of the three filters (from 0.41 before filtering, to ≈0.6 − 0.65 afterwards).
(TIF)

S4 FigImprovement in $\hat{C}$ by *ω*_thresh_.Mean value and standard deviation of the change, $\Delta \hat{C}$, in the Pearson correlation coefficient between the single time frame barcode and the aligned kymograph time average after filtering. Results are averages over the 6400 barcodes and the values of *ω*_thresh_ are in units of *ω*_psf_ (FWHM of the PSF of the system). We see that for the Gaussian and Moving average filters, using 0.5*ω*_psf_ ≤ *ω*_thresh_ ≤ 1.5*ω*_psf_ produces the highest average improvement in the correlation, while for the Window-Sinc filter the optimal value are *ω*_psf_ ≤ *ω*_thresh_ ≤ 2*ω*_psf_.(TIF)

S5 FigFinal (optimal) values for the filter parameters.Distribution of the value of the parameters for the three filters and for *ω*_thresh_ = *ω*_psf_. We observe that *σ*_gaussian_ = 1.7 ± 0.4 pixels, *b* = 3 ± 0.4 pixels and *f*_cut-off_ = 0.18 ± 0.03 pixels^−1^. For quick and simple filtering we suggest the following choice of filter parameters: *σ*_gaussian_ ≈ *σ*_psf_, *b* ≈ 1.5*σ*_psf_ and *f*_cut-off_ ≈ 1/(*πσ*_psf_), for Gaussian, Moving Average and Window-Sinc filtering, respectively. The choice for *f*_cut-off_ follows from a “two sigma rule” of the Fourier transform of a single Gaussian with standard deviation *σ*_psf_, $\phi \left(x\right)=\exp\left[-x^{2}/\left(2\sigma_{\text{psf}}\right)\right]/\sqrt{2\pi \sigma_{\text{psf}}^{2}}$: the Fourier transform of this function is $\Phi \left(f\right)=\int_{-\infty }^{\infty }\exp\left(2\pi ifx\right)\phi \left(x\right)dx=\exp\left[-f^{2}/\left(2S^{2}\right)\right]$ with *S* = 1/(2*πσ*_psf_). Applying the two sigma rule, *f* = *f*_cut-off_ = 2*S*, gives our suggested value for the frequency cut-off for the Window-Sinc filter. The suggested values above are good approximations if one decides to not apply recursive filtering. However, beware that these suggested values may be specific to the present data set and not optimal for other data sets obtained using the competitive binding assay nor for other types of optical DNA maps.(TIF)
