# Supplementary material for: Noise reduction in single time frame optical DNA maps
Source: PLoS One. 2017 Jun 22;12(6):e0179041. doi: 10.1371/journal.pone.0179041 (PMC5480869; doi:10.1371/journal.pone.0179041)
Supplement: S1 Table — From left to right: Is = mean intensity of the signal part of the barcodes (a.u.), where the errors are the standard deviation over different molecules, σs = the standard deviation in intensity for single time frame barcodes around the time-averaged barcode (a.u.), Ibg = mean intensity level of the background (a.u), σbg = standard deviation in the background intensity (a.u), length = average lengths of molecules (pixels), σlength = average of standard deviations in length in a kymograph (pixels). Brackets, 〈…〉, corresponds to an average over different molecules. (PDF) [file pone.0179041.s001.pdf]

S1 Table

	Properties of the barcodes					
**Type of barcode**	$\langle I_s \rangle$	$\langle \sigma_s \rangle$	$\langle I_{\rm bg} \rangle$	$\langle \sigma_{\rm bg} \rangle$	$\langle$length$\rangle$	$\langle \sigma_{\rm length} \rangle$
pUUH	931 (± 29)	47	731	7	348	6
pEC005A	1300 (± 111)	71	789	9	115	4
pEC005B	1244 (± 119)	63	785	8	235	3
