# Supplementary material for: Noise reduction in single time frame optical DNA maps
Source: PLoS One. 2017 Jun 22;12(6):e0179041. doi: 10.1371/journal.pone.0179041 (PMC5480869; doi:10.1371/journal.pone.0179041)
Supplement: S2 Table — Change in C^ between the single time-frame and the aligned kymograph time average after reducing the noise with our method with the three filters (Gaussian, Moving average and Window-Sinc) independently. The values are an average over the three filters and over all time-frame barcodes of the same type (pUUH, pEC005A and pEC005B). C^ust,ta is the correlation between the unfiltered single time-frame (ust) barcode before any filtering and its aligned kymograph time average (ta). C^fst,ta is the correlation between the filtered barcode (fst) and the aligned kymograph time average. We see that in pUUH barcodes (the longest DNAs) the correlation improves slightly more than for the other two barcode types (pEC005A and pEC005B). (PDF) [file pone.0179041.s002.pdf]

S2 Table

**Type of barcode**	$\langle \Delta\hat{C} \rangle = \langle \hat{C}_{fst,ta} - \hat{C}_{ust,ta} \rangle$	$\sigma_{\hat{C}_{fst,ta} - \hat{C}_{ust,ta}}$
pUUH	0.23	0.06
pEC005A	0.15	0.05
pEC005B	0.15	0.04
