# Supplementary figures and images for: Noise reduction in single time frame optical DNA maps
Source: PLoS One. 2017 Jun 22;12(6):e0179041. doi: 10.1371/journal.pone.0179041 (PMC5480869; doi:10.1371/journal.pone.0179041)

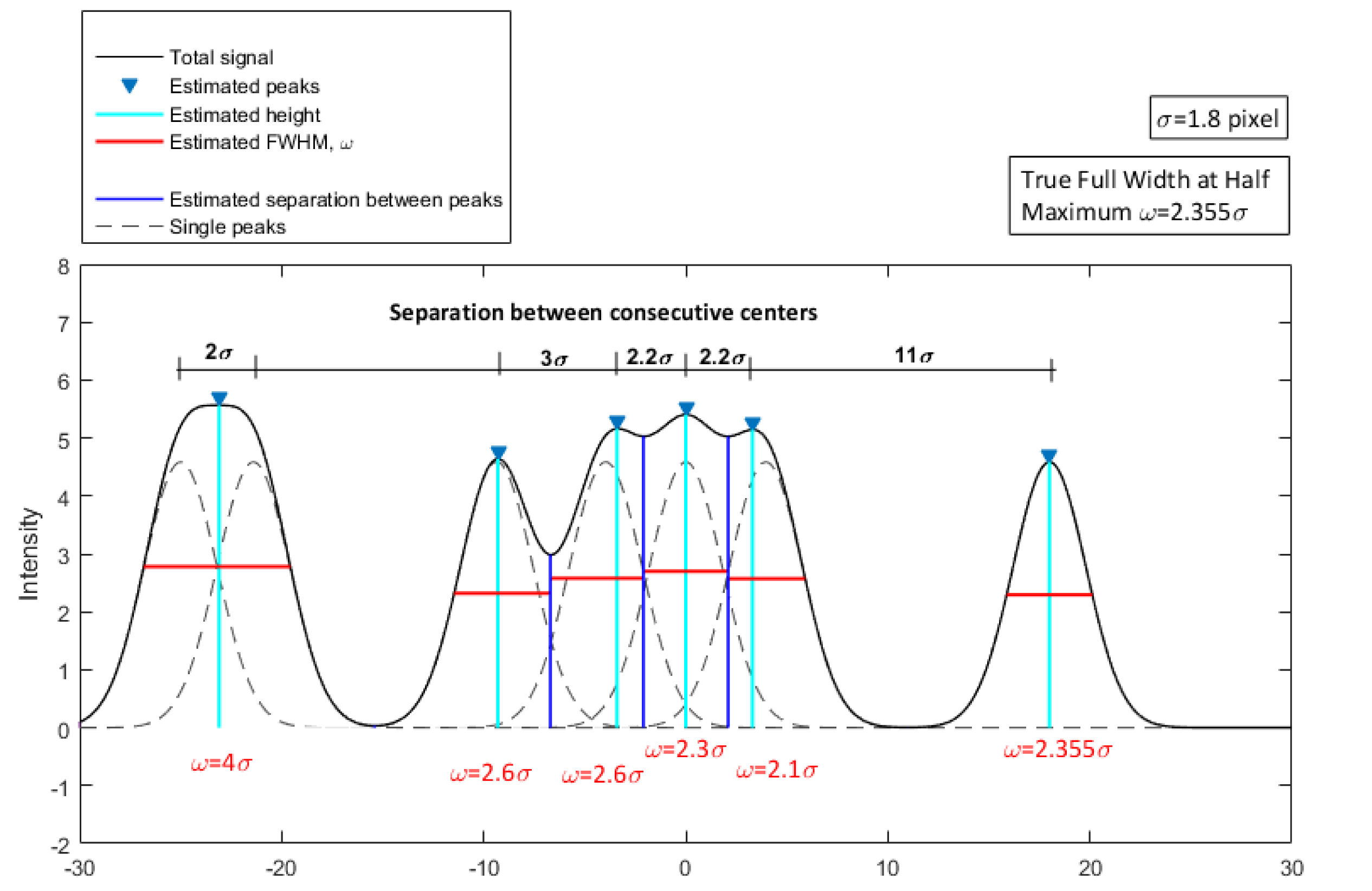

Supplement: S1 Fig — For estimating ω when peaks overlap, which is the case in our densely labeled barcodes, we use an algorithm implemented in Matlab Version 2015b in the function findpeaks(PeakSig, x, ‘WidthReference’, ‘halfheight’), where PeakSig is the intensity signal, x the position (pixels in our case) and ‘WidthReference’, ‘halfheight’ indicates the method for estimating the width of the peaks. In short, this function finds all maxima and estimates the full width at half maximum as follows: 1) Find all local maxima (peaks) (blue triangles). 2) The height of a peak is defined as the vertical distance between its maximum value and 0 (light blue line). 3) Detect the local minima on both sides of the peak. If a local minimum is not of intensity 0, draw a vertical line from that local minimum to 0 (blue line). 4) The estimated FWHM, ω, is then the distance, measured at half height of the peak, between the peak signal, or the drawn vertical line, from one side of the maximum of the peak to the other (red line). As shown in the examples in the figure, this method is rather successful at estimating the “true” width, i.e. 2.355σ, in a scenario of overlapping Gaussians. (TIF) [file pone.0179041.s003.tif]

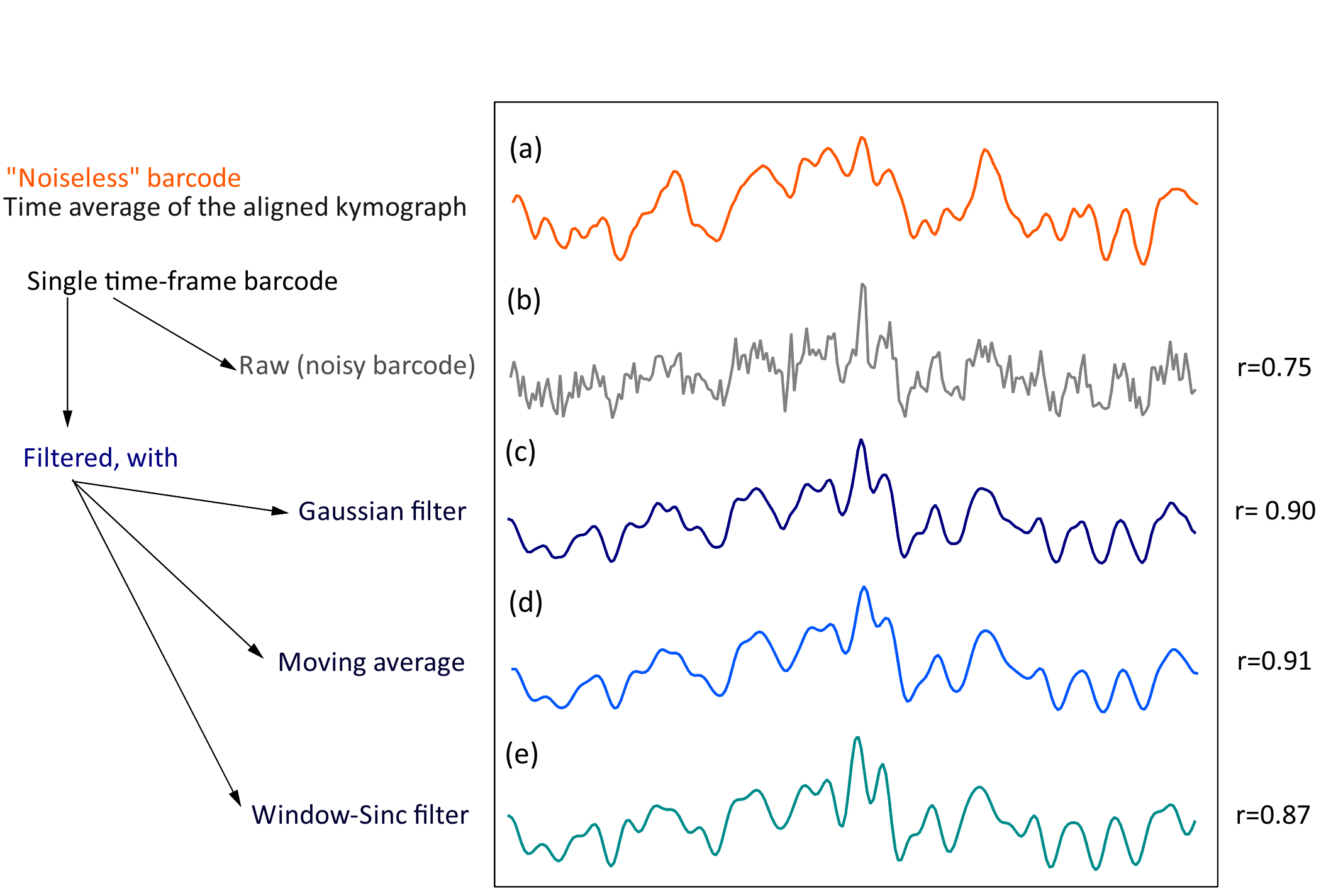

Supplement: S2 Fig — (a) Time average of the aligned kymograph (“noiseless” barcode). (b) Frame 100 (measured during 0.1s) extracted from the kymograph (noisy barcode). The three barcodes (c)-(e) are the result of using our method to reduce the noise with: (c) a Gaussian filter, (d) a Moving average filter, (e) a Window-Sinc filter. The Pearson correlation coefficient between the time average of the aligned kymograph and the single frame barcode improves by ≈0.15 points after filtering, with any of the three filters (from 0.75 before filtering, to ≈0.9 afterwards). (TIF) [file pone.0179041.s004.tif]

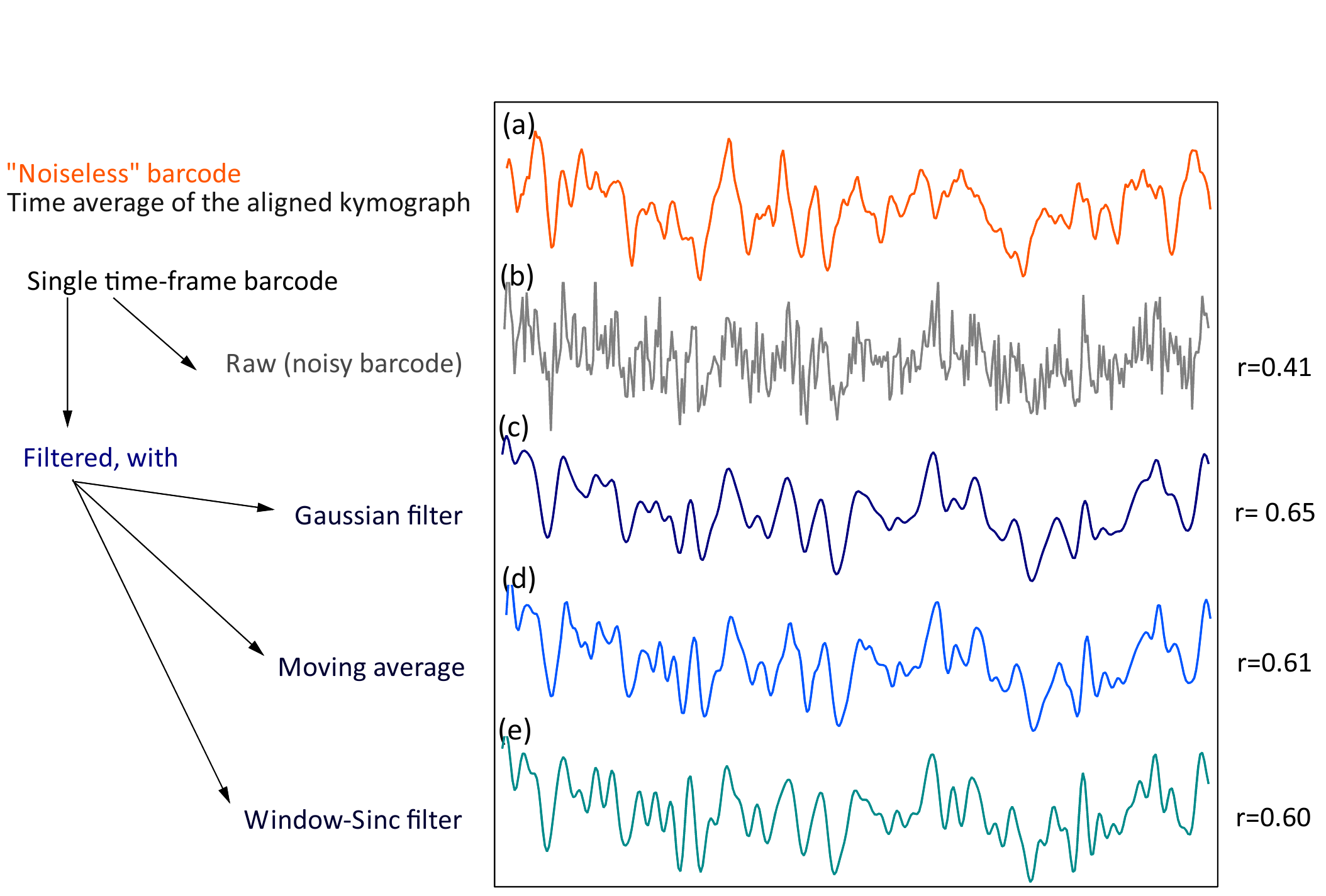

Supplement: S3 Fig — (a) Time average of the aligned kymograph (“noiseless” barcode). (b) Frame 100 (measured during 0.1s) extracted from the kymograph (noisy barcode). The barcodes (c)-(e) are the result of using our method to reduce the noise with: (c) a Gaussian filter, (d) Moving average filter, (e) a Window-Sinc filter. The Pearson correlation coefficient between the time average of the aligned kymograph and the single frame barcode improves in ≈0.2 points after filtering, with any of the three filters (from 0.41 before filtering, to ≈0.6 − 0.65 afterwards). (TIF) [file pone.0179041.s005.tif]

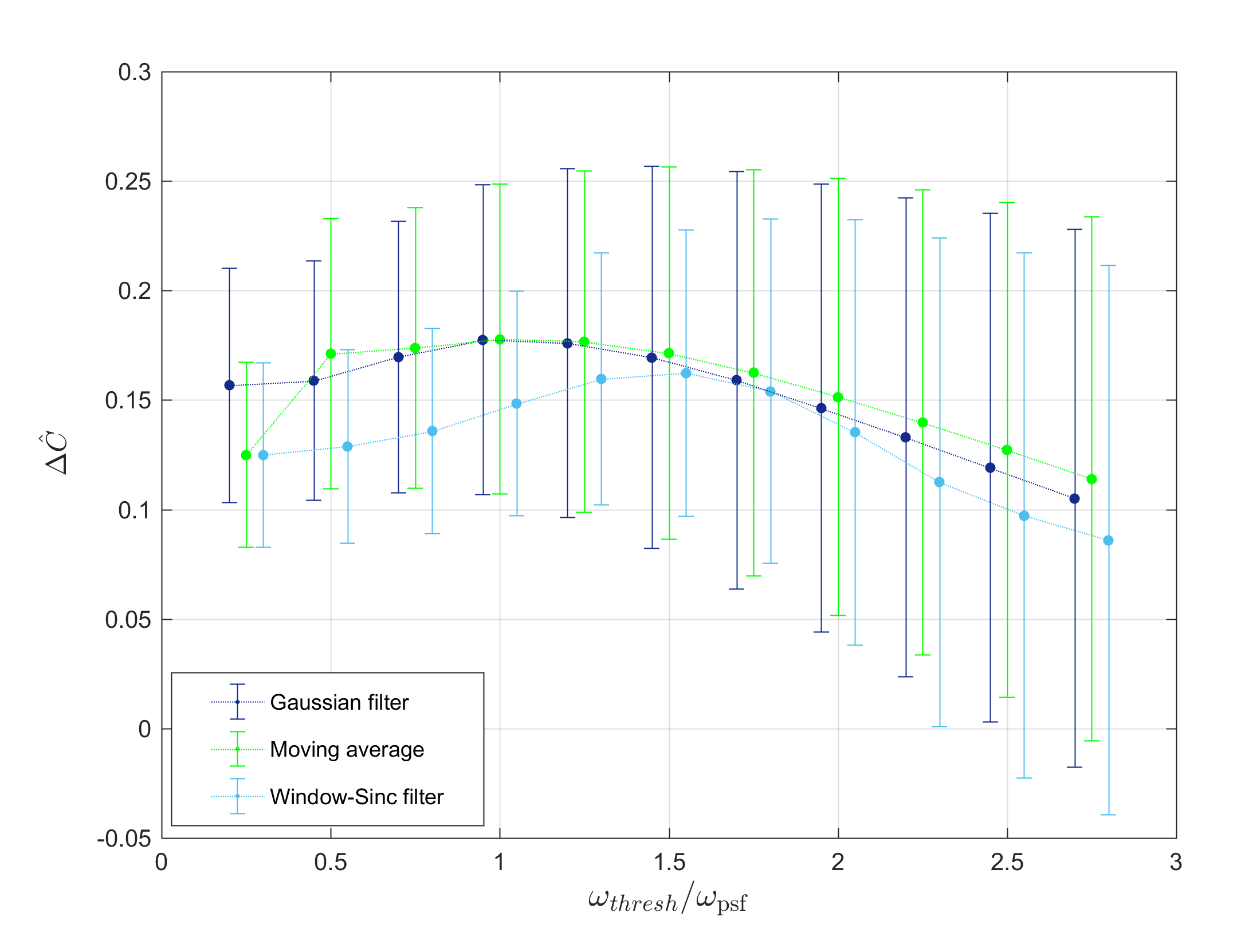

Supplement: S4 Fig — Mean value and standard deviation of the change, ΔC^, in the Pearson correlation coefficient between the single time frame barcode and the aligned kymograph time average after filtering. Results are averages over the 6400 barcodes and the values of ωthresh are in units of ωpsf (FWHM of the PSF of the system). We see that for the Gaussian and Moving average filters, using 0.5ωpsf ≤ ωthresh ≤ 1.5ωpsf produces the highest average improvement in the correlation, while for the Window-Sinc filter the optimal value are ωpsf ≤ ωthresh ≤ 2ωpsf. (TIF) [file pone.0179041.s006.tif]

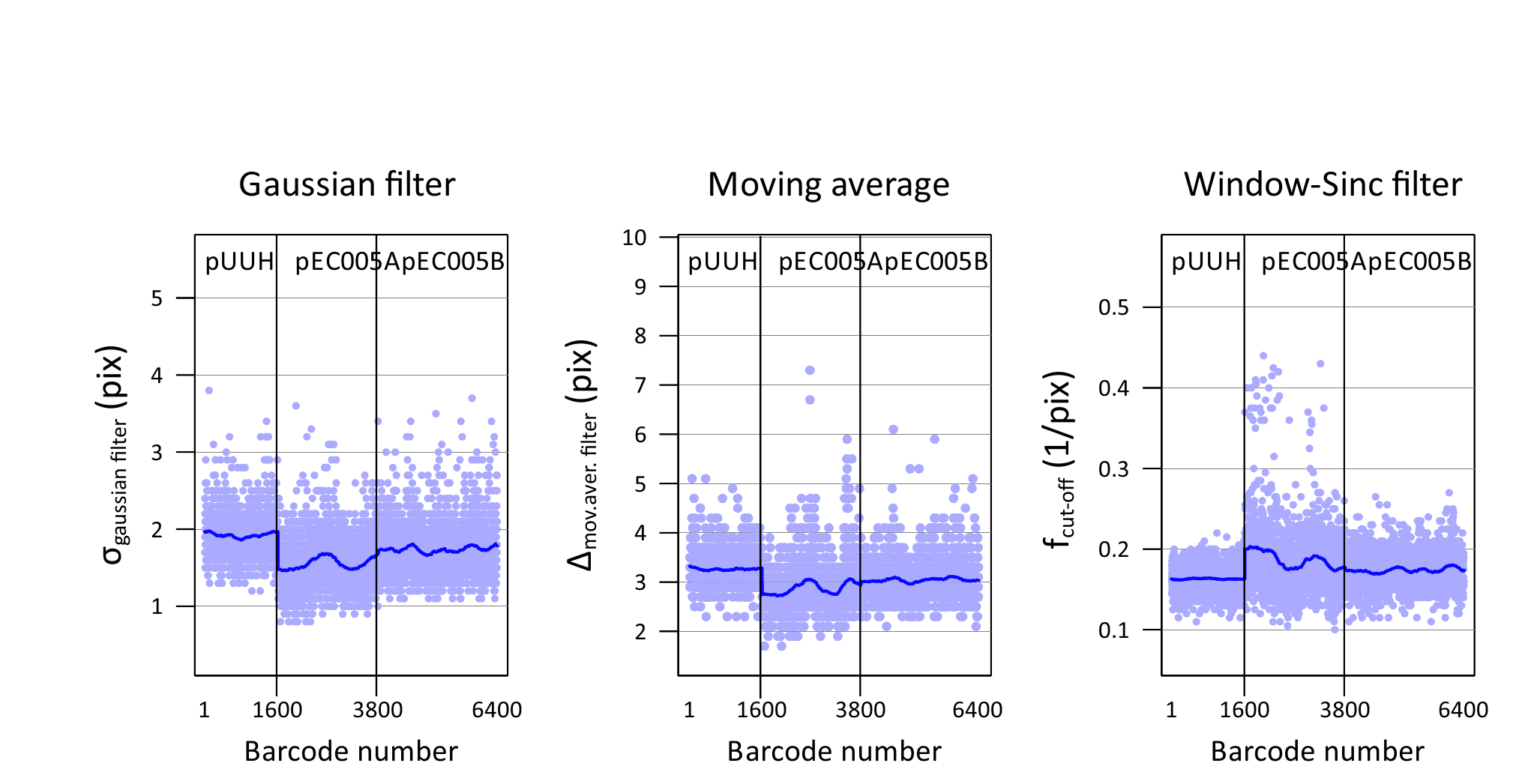

Supplement: S5 Fig — Distribution of the value of the parameters for the three filters and for ωthresh = ωpsf. We observe that σgaussian = 1.7 ± 0.4 pixels, b = 3 ± 0.4 pixels and fcut-off = 0.18 ± 0.03 pixels−1. For quick and simple filtering we suggest the following choice of filter parameters: σgaussian ≈ σpsf, b ≈ 1.5σpsf and fcut-off ≈ 1/(πσpsf), for Gaussian, Moving Average and Window-Sinc filtering, respectively. The choice for fcut-off follows from a “two sigma rule” of the Fourier transform of a single Gaussian with standard deviation σpsf, ϕ(x)=exp[-x2/(2σpsf)]/2πσpsf2: the Fourier transform of this function is Φ(f)=∫-∞∞exp(2πifx)ϕ(x)dx=exp[-f2/(2S2)] with S = 1/(2πσpsf). Applying the two sigma rule, f = fcut-off = 2S, gives our suggested value for the frequency cut-off for the Window-Sinc filter. The suggested values above are good approximations if one decides to not apply recursive filtering. However, beware that these suggested values may be specific to the present data set and not optimal for other data sets obtained using the competitive binding assay nor for other types of optical DNA maps. (TIF) [file pone.0179041.s007.tif]
